# Venous thromboembolism prophylaxis on flights

**DOI:** 10.1590/1677-5449.010817

**Published:** 2018

**Authors:** Marcos Arêas Marques, Marilia Duarte Brandão Panico, Carmen Lucia Lascasas Porto, Ana Letícia de Matos Milhomens, Juliana de Miranda Vieira

**Affiliations:** 1 Universidade do Estado do Rio de Janeiro (UERJ), Hospital Universitário Pedro Ernesto, Rio de Janeiro, RJ, Brasil.

**Keywords:** deep vein thrombosis, pulmonary embolism, air travel, aerospace medicine, prophylaxis

## Abstract

Civil aviation has seen a steady increase in the number of scheduled flights over the last ten years and, as a result, more passengers are traveling by air. This has been associated with an increase in flight-related diseases, especially on long-haul flights. One of the most feared complications during flights is venous thromboembolism (VTE), but its true incidence is difficult to measure because of a lack of consensus on elements such as the definition of how long after landing a VTE can be considered to be related to a flight and even how long a flight must last to be considered of long duration. There has been much discussion of the pathophysiological mechanisms of flight-related VTE, of which passengers are at greatest risk, and of what prophylactic measures can be adopted safely and effectively. The purpose of this review is to clarify these points and describe current consensual conduct.

## INTRODUCTION

 It is estimated that, worldwide, approximately 3.5 billion people traveled by air in 2015, which was an increase of 6.8% over the previous year. 1 Over the last 10 years, with the exception of 2016, in Brazil the number of passengers on scheduled flights has been increasing in line with the global growth trend. According to the Brazilian Civil Aviation Authority (ANAC), in 2016 around 115 million Brazilians traveled on domestic and international flights. 2 In parallel with this growth in the number of passengers, there has, naturally, been an increase in medical conditions and diseases related to flights, especially those considered long duration or long distance. In general, the conditions most frequently reported are: hypoxia, transmission of infectious and contagious diseases, jet lag, anxiety attacks, and venous thromboembolism (VTE). 3 The growing number of fatal flight-related VTE cases has attracted the attention of the general public and the non-specialist press, in addition to health professionals, who attempt to reach consensus on prophylactic measures for this complication. 

## METHODOLOGY

 This is a literature review study that analyzed publications available on the PubMed database for the period spanning 2001 to 2017. Searches were run using the following keywords: deep vein thrombosis, pulmonary embolism, air travel, aerospace medicine, and prophylaxis. The criterion for inclusion of articles in the study was at least one of these keywords. 

## RESULTS AND DISCUSSION

### Epidemiology

 It is very difficult to define which VTE episodes have a direct relationship with flights, since there is no established consensus on the maximum interval of time between landing and diagnosis of VTE that can be defined as indicating that the two are associated. 3 The first studies were based on clinical data alone and did not confirm diagnoses with laboratory tests or ultrasound examination, and there is a large range of variation between studies in terms of screening for the disease and definitions of what constitutes a long-haul flight. 1 ^,^ 3 Another factor that makes a more consistent epidemiological analysis difficult is that some studies only investigate the incidence of deep venous thrombosis (DVT), others pulmonary embolism (PE), and yet others cover both diseases. 

 Incidence and prevalence rates of VTE reported in studies also vary depending on the methods used for diagnosis and whether cases were sought actively, for example in asymptomatic patients, or whether only symptomatic patients who seek medical care after a flight were considered. However, there does indeed appear to be a direct relationship between flight duration and VTE episodes, which, in the majority of cases, occur within the first 2 weeks after landing, with a mean interval of 4 days, while the risk is present for 4 weeks. 3 ^,^ 4

 A meta-analysis published in 2009, with 14 studies involving 4,055 episodes of DVT demonstrated that overall grouped relative risk is 2.8 times (95% confidence interval: 2.2-3.7) for long duration flights. Additionally, the absolute risk of symptomatic DVT, in the general population, during the eight weeks following a long-haul flight is one in every 4,500 flights. 4 These figures have not led to widespread adoption of universal measures for VTE prophylaxis; but the risk can be significantly higher among passengers considered at high risk of development of DVT and/or PE. A cohort study with 7,592 employees of large corporations who fly regularly and were followed for a mean period of 4.4 years found that the risk of VTE can be as much as 20 times greater in passengers who have recently undergone surgery and up to 18 times greater in passengers with a diagnosis of active cancer. 4 In these cases, these passengers would probably benefit from prophylactic measures to prevent VTE. 

### Risk factors related to planes and flights

#### Hypoxia

 For economic reasons, on the majority of commercial flights atmospheric pressure inside the plane is maintained similar to that found at altitudes of 1,800 to 2,400 meters above sea level, because maintaining pressure higher than this demands greater fuel consumption since it increases the weight of the aircraft. 3 The prolonged hypoxia resulting from this can provoke activation of the extrinsic coagulation pathway via thromboplastin-carrying microparticles. 5

#### Position during the flight

 Venous stasis provoked by the seated position maintained for long periods of time with little space between the rows to enable regular movement of feet and lower limbs, especially in economy class, can also be a trigger factor of the coagulation cascade during a flight 3 ^,^ 5 and, consequently, of development of VTE. 

#### Dehydration

 Low relative air humidity inside the airplane during a flight can cause dehydration, hemoconcentration, and hyperviscosity of the blood, making development of VTE more likely. Additionally, dehydration can be exacerbated by regular consumption of alcoholic beverages, coffee, and tea, which are drinks known to induce diuresis. 3

 In isolation, it is unlikely that hypoxia, stasis, or dehydration are enough to provoke DVT and/or PE in long-haul passengers, but the combination of at least two of these three factors may be sufficient for development of VTE. 3 ^,^ 5

#### Flight duration

 There is no universal consensus on what length of flight should be defined as of long duration, but there is a clear relationship between development of VTE and flights lasting longer than 6 hours. The risk of VTE is 2.3 times greater in long flights than short flights and risk increases by 26% for every additional 2 hours of flight time 1 ^,^ 3 The estimated risk of fatal PE is 0.5/10^6^ for flights lasting more than 3 hours and 1.3/10^6^ for flights exceeding 8 hours’ duration. 6

#### Class and location of seat

 Despite the well-known term “economy class syndrome” being used systematically as a synonym for flight-related VTE, there is very little difference between passengers who travel economy, business, and first class. 3 ^,^ 7 However, passengers who travel in window seats are at double the risk of VTE than passengers traveling in aisle seats, especially for obese passengers (body mass index, BMI ≥ 30 kg/m^2^). 7

#### Passenger-related risk factors

#####  Oral contraceptives, Hormone Replacement Therapy (HRT), and pregnant women 

 The Multiple Environmental and Genetic Assessment (MEGA) 8 study analyzed 1,906 flight or land passengers less than 70 years old, who had a first DVT or PE episode, at six regional anticoagulation clinics in Holland. The results showed that women on oral contraceptives have up to a 40 times greater chance of developing DVT on long-haul flights. Additionally, female sex is considered an independent risk factor for PE on this type of flight. 3

 The absolute estimated risk of flight-related VTE among women on oral contraceptives is one in every 259 flights, while for women on HRT the risk is one in every 405 flights and it is one in every 109 flights for pregnant women. 4

##### Obesity

 Overweight passengers (BMI: 25-30 kg/m^2^) and, especially, obese passengers (BMI ≥ 30 kg/m^2^) who travel in window seats are at greater risk of developing VTE on long-haul flights (OR: 6.1). 3 ^,^ 7 ^,^ 9

##### Recent surgery

 The risk of developing VTE increases almost 20 times among passengers who have recently undergone surgery, when compared to passengers who have not (OR: 19.8). 4

##### Cancer

 In general, cancer increases the incidence of VTE by four to seven times, 10 but the risk can be as much as 18 times greater on long haul flights. 4

##### Thrombophilias

 There are several controversies related to the importance of acquired or hereditary thrombophilias in development of long-haul flight-related VTE. However, the MEGA study found that long-haul passengers with factor V Leiden had around an eight times greater risk of developing DVT than passengers who do not. 8

##### Others

 Many other diseases or inherent passenger characteristics may be associated with increased incidence of VTE on long-duration flights. These include extremes of stature, recent trauma with immobilization, age over 40 years, advanced chronic venous disease, anxiety, prior or family history of VTE, and congestive heart failure. 1 ^,^ 3 ^,^ 4 ^,^ 8 ^,^ 9 ^,^ 11

#### Prophylaxis

##### General measures

 Control of air humidity with humidification by air-conditioning may be an effective protective measure against dehydration and, as a result, against hemoconcentration and resulting increases in blood viscosity, which could induce VTE development. 3 Furthermore, patients should be encouraged to ensure a regular intake of water or juice rather than alcoholic beverages, coffee, or tea, which induce diuresis. Other simple measures that can help to prevent flight-related VTE include encouraging passengers to move their feet and legs, with plantar dorsiflexion, for example, and advising passengers to choose aisle seats, which makes it easier to walk inside the plane during the flight. 

 The ninth edition of the American College of Chest Physicians’ (ACCP) antithrombotic and thrombosis prevention guidelines, from 2012, 11 suggests the following for passengers considered at high risk of VTE on long haul flights (previous VTE episode, recent surgery or trauma, active cancer, pregnancy, taking estrogen, advanced age, limited mobility, obesity, or thrombophilias): walking, calf muscle exercises, and an aisle seat (evidence level 2C). 

##### Graduated Elastic Compression Stockings (GECS)

 Theoretically, wearing GECS during flights increases venous return from the lower limbs and, thus, reduces venous stasis provoked by the immobility caused by remaining in a seated position for long periods of time, thereby reducing the risk of VTE. A recent Cochrane review evaluated 11 randomized studies, with a total of 2,906 passengers (1,273 high-risk patients), on flights lasting more than 5 hours, comparing those who wore MECG on both legs, with those who did not wear them at all, and those who wore them on just one leg. It concluded that there is high quality evidence of a reduction in the number of asymptomatic DVTs and low quality evidence of a relationship with reduced volume of lower limb edema. There was also moderate quality evidence of reduction in superficial venous thrombosis among these passengers, 12 confirming the findings of the LONFLIT-2 and 5 studies. 13 ^,^ 14 Furthermore, GECS may be a good prophylactic option for passengers with contraindications against drug-based prophylaxis or at a high risk of bleeding. 9 The ninth edition of ACCP’s antithrombotic and thrombosis prevention guidelines suggests that passengers on long-haul flights who are at high risk of development of VTE (previous VTE episode, recent surgery or trauma, active cancer, pregnancy, taking estrogen, advanced age, limited mobility, obesity, or thrombophilias) should wear below-the-knee MECG providing 15 to 30 mmHg pressure at the ankle during the flight (evidence level 2C). For all other long-haul passengers, the recommendation is not to wear MECG (evidence level 2C). 11

#### Pharmacological prophylaxis

##### Platelet antiaggregants

 Platelet antiaggregants did not prove to be an effective measure for primary or secondary long-haul flight-related VTE prophylaxis. 1 ^,^ 3 ^,^ 9 The ninth edition of the ACCP guidelines recommends that aspirin should not be used as a prophylactic measure in these cases (evidence level 2C) 11 because of the clinically relevant increased risk of bleeding. 9

##### Low Molecular Weight Heparin (LMWH)

 There are doubts with relation to evidence of whether LMWH may be effective for prevention of long-haul flight-related VTE. 9 The LONFLIT-3 study suggests that the risk of VTE can be practically eliminated by taking LMWH, 1 but there is not enough evidence to confirm its indiscriminate and universal use in this situation. 15 In general, pharmacological prophylaxis with LMWH on long-haul flights should be reserved for passengers considered at high risk of development of VTE, and the decision on whether or not to employ it should be made after measurement of the risks and benefits for each passenger individually. 11 ^,^ 15 Enoxaparin at a dosage of 1 mg/kg, via subcutaneous injection, 2 to 4 hours before departure, can reduce the risk of VTE on long-haul flights. 3 There is indirect evidence of increased risk of bleeding related to use of pharmacological VTE prophylaxis on long-haul flights. 6 Additionally, the financial costs of using anticoagulants for VTE prophylaxis in these passengers should also be taken into account. 6

##### Direct Oral Anticoagulants (DOACSs)

 There are certain considerations that should be taken into account with relation to use of DOACSs for pharmacological long-haul flight-related VTE prophylaxis. This group of medications, which includes direct factor Xa inhibitors (in Brazil, Rivaroxaban, apixaban, and edoxaban) or thrombin inhibitors (in Brazil, dabigatran), has certain relevant characteristics that will possibly change current prophylactic conduct, as real-life data are published. Their short half-lives, rapid onsets of action, and posologies via oral route may make them the drugs of choice in the relatively near future. However, to date, studies have not been conducted that confirm their efficacy and safety for VTE prophylaxis on long-haul flights. 1

## CONCLUSIONS

 There are still many doubts with relation to which profiles of passengers can truly benefit from prophylactic measures against development of VTE related to long-haul flights. Additionally, there is still no universal consensus with relation to which behavioral, physical, or pharmacological measures should be adopted to protect each particular passenger. This is compounded by the fact that there is no epidemiological definition of which VTE episodes can be related to long-haul flights or how many hours in the air qualify a flight as a long-haul flight. Therefore, all passengers should be assessed individually, weighing up the risks and benefits of adopting each of the different measures for VTE prophylaxis on long-haul flights. 
