# Supplementary material for: Venous thromboembolism prophylaxis on flights
Source: J Vasc Bras. 2018 Jul-Sep;17(3):215–9. doi: 10.1590/1677-5449.010817 (PMC6326126; doi:10.1590/1677-5449.010817)
Supplement: Translation

# Translation: Profilaxia do tromboembolismo venoso em viagens aéreas

**Authors:** Marcos Arêas Marques, Marilia Duarte Brandão Panico, Carmen Lucia Lascasas Porto, Ana Letícia de Matos Milhomens, Juliana de Miranda Vieira

**Affiliations:** 1 Universidade do Estado do Rio de Janeiro (UERJ), Hospital Universitário Pedro Ernesto, Rio de Janeiro, RJ, Brasil.

**Keywords:** trombose venosa profunda, embolia pulmonar, viagem aérea, medicina aeroespacial, profilaxia

## Abstract

A aviação civil vem apresentando aumento progressivo do número de voos regulares nos últimos 10 anos e, em função disso, mais passageiros estão sendo transportados em viagens aéreas (VAs). Associado a isso, há um aumento das doenças relacionadas às VAs, especialmente naquelas de longa duração. Uma das complicações mais temidas dos voos é o tromboembolismo venoso (TEV), mas a sua real incidência é de difícil mensuração devido à falta de consenso sobre, por exemplo, quanto tempo após o pouso podemos considerar que o TEV possa estar relacionado à VA realizada ou mesmo quanto tempo de voo pode ser considerado como de longa duração. Muito tem se discutido sobre os mecanismos fisiopatológicos do TEV relacionado às VAs, quais passageiros são os de maior risco e quais medidas profiláticas podemos adotar com segurança e eficácia. O objetivo desta revisão é esclarecer esses pontos e as condutas consensuais atuais.

## INTRODUÇÃO

 Estima-se que, no ano de 2015, aproximadamente 3,5 bilhões de pessoas fizeram viagens aéreas (VAs) em todo o mundo, um crescimento de 6,8% em relação ao ano anterior 1 . No Brasil, nos últimos 10 anos, com exceção de 2016, o número de passageiros em voos comerciais vem acompanhando a tendência de crescimento mundial. Segundo a Agência Nacional de Aviação Civil (ANAC), em 2016, cerca de 115 milhões de brasileiros foram transportados em voos aéreos nacionais e internacionais 2 . Em paralelo ao crescimento do número de passageiros, houve, naturalmente, um aumento de intercorrências médicas e de doenças relacionadas às VAs, especialmente aquelas consideradas de longa duração ou distância. De forma geral, as intercorrências mais relatadas são: hipóxia, transmissão de doenças infectocontagiosas, *jet lag,* crise de ansiedade e tromboembolismo venoso (TEV) 3 . O número crescente de casos fatais de TEV relacionado às VAs tem chamado atenção do público geral e da imprensa leiga, além dos profissionais de saúde, que tentam buscar condutas consensuais para a profilaxia dessa complicação. 

## METODOLOGIA

 Trata-se de um estudo de revisão de literatura que considerou as publicações disponíveis na base de dados bibliográficos PubMed no período de 2001 a 2017. A pesquisa utilizou as seguintes palavras-chave: *deep vein thrombosis, pulmonary embolism, air travel, aerospace medicine, prophylaxis*. O critério de inclusão de artigos no estudo foi contemplar pelo menos uma das palavras-chave. 

## RESULTADOS E DISCUSSÃO

### Epidemiologia

 Existe uma grande dificuldade em definir quais episódios de TEV podem ter relação direta com as VAs, pois não existe consenso estabelecido sobre o intervalo máximo de tempo entre o pouso do avião e o diagnóstico do TEV que caracterize a associação de ambos 3 . Os primeiros estudos foram baseados em dados clínicos isolados, sem confirmação laboratorial ou ecográfica, além de haver uma grande variação nos estudos com relação ao tempo de rastreamento da doença e à definição de voos de longa distância 1 ^,^ 3 . Outro fato que dificulta uma análise epidemiológica mais consistente é que alguns trabalhos relatam exclusivamente a incidência de trombose venosa profunda (TVP), outros apenas a de embolia pulmonar (EP), enquanto alguns englobam ambas as doenças. 

 A incidência e a prevalência de TEV nos estudos também variam de acordo com o método utilizado para o diagnóstico e se este foi feito através de busca ativa, por exemplo, em pacientes assintomáticos, ou se foi feito apenas naqueles pacientes sintomáticos que procuraram auxílio médico após o voo. Mas, de fato, parece existir uma relação direta com o tempo de duração do voo, e os episódios de TEV, na maioria das vezes, ocorrem nas primeiras duas semanas após a aterrisagem, com média de quatro dias, e o risco permanece por quatro semanas 3 ^,^ 4 . 

 Uma metanálise publicada em 2009, com 14 estudos envolvendo 4.055 episódios de TVP, demonstrou que o risco relativo global agrupado é de 2,8 vezes (intervalo de confiança de 95%: 2,2-3,7) em viagens aéreas prolongadas. Além disso, o risco absoluto de TVP sintomática, na população geral, nas oito semanas seguintes a um voo de longa duração, é de um a cada 4.500 voos 4 . Esses números não estimulariam as condutas universais e disseminadas de profilaxia do TEV; porém, o risco pode aumentar de forma significativa em passageiros considerados de alto risco para o desenvolvimento de TVP e/ou EP. Um estudo de coorte com 7.592 funcionários de grandes corporações que viajavam regularmente, acompanhados em média por cerca de 4,4 anos, demonstrou que o risco de TEV pode aumentar em até 20 vezes em passageiros que foram submetidos a cirurgia recentemente e até 18 vezes em passageiros com diagnóstico de câncer ativo 4 . Nesses casos, portanto, esses passageiros provavelmente podem se beneficiar das medidas profiláticas para o TEV. 

###  Fatores de riscos relacionados ao avião e à viagem aérea 

#### Hipóxia

 Por uma questão econômica, a maioria dos voos comerciais mantém, no interior do avião, uma pressão atmosférica (PA) similar à encontrada em altitudes situadas entre 1.800 e 2.400 metros acima do nível do mar, pois manter a PA mais elevada que isso requer maior consumo de combustível devido ao aumento do peso da aeonave 3 . A hipóxia prolongada resultante desse fato pode provocar a ativação da via extrínseca da coagulação através de micropartículas portadoras de fator tecidual 5 . 

#### Posição de viagem

 A estase venosa provocada pela posição sentada, com pouco espaço entre as fileiras que permitam mobilização regular dos pés e dos membros inferiores, especialmente na classe econômica, por longos períodos de tempo, também pode ser um gatilho para a ativação da cascata de coagulação em VA 3 ^,^ 5 e, consequentemente, para o desenvolvimento de TEV. 

#### Desidratação

 A baixa umidade relativa do ar ambiente dentro de uma aeronave durante a VA pode levar a desidratação, hemoconcentração e hiperviscosidade sanguínea, o que favorece o desenvolvimento de TEV. Além disso, a desidratação pode ser potencializada pelo consumo regular de bebidas alcoólicas, café e chá, que são bebidas sabidamente indutoras da diurese 3 . 

 Isoladamente, tanto a hipóxia quanto a estase e a desidratação provavelmente não seriam suficientes para provocar TVP e/ou EP nos passageiros de VA de longa duração, mas a conjunção de pelo menos dois desses três fatores poderia ser suficiente para o desenvolvimento de TEV 3 ^,^ 5 . 

#### Duração do voo

 Não existe um consenso universal sobre qual é o tempo de voo que possa ser caracterizada como de longa duração, mas existe uma clara relação entre o desenvolvimento de TEV e voos com duração maior que seis horas. O risco de TEV aumenta 2,3 vezes em voos longos quando comparados a voos curtos, sendo que o risco cresce 26% a cada duas horas adicionais de voo 1 ^,^ 3 . O risco estimado de EP fatal é de 0,5/10^6^ para voos acima de três horas e de 1,3/10^6^ para voos acima de oito horas de duração 6 . 

#### Tipo de classe e localização do assento

 Apesar do termo consagrado “síndrome da classe econômica” ser usado sistematicamente como sinônimo de TEV relacionado às VAs, existe muito pouca diferença de risco entre os passageiros que viajam de classe econômica, primeira classe ou classe executiva 3 ^,^ 7 . Porém, para os passageiros que viajam em assentos situados ao lado da janela, o risco de TEV aumenta em duas vezes quando comparados aos passageiros que viajam em assentos situados ao lado do corredor, especialmente se o passageiro for obeso (índice de massa corporal, IMC ≥ 30 kg/m^2^) 7 . 

#### Fatores de risco relacionados aos passageiros

#####  Anticoncepcionais Orais (ACOs), Terapia de Reposição Hormonal (TRH) e gestantes 

 O estudo *The Multiple Environmental and Genetic Assessment* (MEGA) 8 avaliou 1.906 passageiros de VAs ou terrestres, com menos de 70 anos de idade, em seu primeiro episódio de TVP ou EP, em seis clínicas regionais de anticoagulação na Holanda. Os resultados mostraram que as mulheres em uso de ACOs têm até 40 vezes mais chances de desenvolver TVP em VAs de longa duração. Além disso, o sexo feminino é considerado um fator de risco independente para EP nesse tipo de VA 3 . 

 O risco absoluto estimado de TEV para mulheres em VA em uso de ACOs é de um para cada 259 voos, enquanto para mulheres em uso de TRH é de um para cada 405 voos e para gestação é de um para cada 109 voos 4 . 

##### Obesidade

 Os passageiros com sobrepeso (IMC: 25-30 kg/m^2^) e, especialmente, os obesos (IMC ≥ 30 kg/m^2^) que viajam sentados em assentos ao lado da janela têm maior risco de desenvolver TEV em VA de longa duração (OR: 6,1) 3 ^,^ 7 ^,^ 9 . 

##### Cirurgia recente

 O risco de desenvolvimento de TEV aumenta em quase 20 vezes nos passageiros submetidos a cirurgia recentemente quando comparados a passageiros que não foram submetidos (OR: 19,8) 4 . 

##### Câncer

 As neoplasias, de forma geral, aumentam a incidência de TEV em quatro a sete vezes 10 , mas o risco pode aumentar em até 18 vezes em VA de longa duração 4 . 

##### Trombofilias

 Existem muitas controvérsias quanto à importância das trombofilias adquiridas ou hereditárias no desenvolvimento do TEV relacionado às VAs de longa duração. Porém, o estudo MEGA atribui aos passageiros desse tipo de VA, portadores do fator V de Leiden, um risco cerca de oito vezes maior de desenvolvimento de TVP quando comparados aos passageiros não portadores 8 . 

##### Outros

 Diversas outras doenças ou características inerentes ao passageiro podem estar associadas ao aumento da incidência de TEV em VA de longa duração. Entre elas, podemos citar extremos de estatura, trauma recente com imobilização, idade superior a 40 anos, doença venosa crônica avançada, ansiedade, história prévia ou familiar de TEV e insuficiência cardíaca congestiva 1 ^,^ 3 ^,^ 4 ^,^ 8 ^,^ 9 ^,^ 11 . 

#### Profilaxia

##### Medidas gerais

 O controle da umidade do ar ambiente com umidificação através do ar-condicionado pode ser uma medida protetora efetiva contra a desidratação e, portanto, contra a hemoconcentração e o aumento da viscosidade sanguínea resultante, que pode induzir o desenvolvimento de TEV 3 . Além disso, deve-se estimular o paciente a realizar ingestão regular de água ou sucos em vez de bebidas alcoólicas, café ou chá, que são indutores da diurese. Outra medida simples que pode auxiliar na profilaxia do TEV associado às VAs é o incentivo à movimentação ativa de pés e pernas, como a dorsiflexão plantar, e a orientação para que os passageiros escolham o assento situado ao lado do corredor, que facilita a deambulação no interior da aeronave durante o voo. 

 A nona edição das diretrizes de terapia antitrombótica e prevenção de trombose do Colégio Americano de Pneumologia (ACCP), de 2012 11 , sugere para passageiros considerados de alto risco para TEV em VA de longa distância (episódio de TEV prévio, cirurgia recente ou trauma, neoplasia em atividade, gestação, uso de estrogênio, idade avançada, mobilidade limitada, obesidade ou trombofilias): deambulação, exercícios musculares das panturrilhas e escolha de assento situado próximo ao corredor (nível de evidência 2C). 

##### Meias Elásticas de Compressão Graduada (MECGs)

 O uso de MECGs durante voos de longa duração, teoricamente, aumentaria o retorno venoso de membros inferiores e, portanto, diminuiria a estase venosa provocada pela imobilidade causada pela posição sentada por longos períodos de tempo, diminuindo, consequentemente, o risco de TEV. Uma revisão recente da Cochrane avaliou 11 estudos randomizados, com total de 2.906 passageiros (1.273 de alto risco), em voos acima de cinco horas de duração, comparando o uso de MECG em ambas as pernas ao não uso ou ao uso em apenas uma das pernas. Concluiu que existe evidência de alta qualidade quanto à redução do número de TVPs assintomáticas e de baixa qualidade em relação à diminuição do volume do edema de membros inferiores. Houve ainda evidência de moderada qualidade quanto à redução de trombose venosa superficial nesses passageiros 12 , corroborando os achados dos estudos LONFLIT-2 e 5 13 ^,^ 14 . Além disso, as MECGs poderiam ser uma boa opção de profilaxia nos passageiros com contraindicação ao uso de farmacoprofilaxia ou com alto risco de sangramento 9 . A nona edição das diretrizes de terapia antitrombótica e prevenção de trombose do ACCP sugere para passageiros de VA de longa distância e de alto risco para desenvolvimento de TEV (episódio de TEV prévio, cirurgia recente ou trauma, neoplasia em atividade, gestação, uso de estrogênio, idade avançada, mobilidade limitada, obesidade ou trombofilias) o uso de MECG abaixo do joelho, com 15 a 30 mmHg de pressão ao nível do tornozelo durante a viagem (nível de evidência 2C). Para todos os outros passageiros de voos de longa duração, sugere contra o uso de MECG (nível de evidência 2C) 11 . 

#### Profilaxia farmacológica

##### Antiagregantes plaquetários

 O uso de antiagregantes plaquetários não se mostrou uma medida eficaz na profilaxia primária ou secundária do TEV relacionado às VAs de longa duração 1 ^,^ 3 ^,^ 9 . A nona edição do ACCP sugere contra o uso de aspirina como medida profilática nesses casos (nível de evidência 2C) 11 devido ao aumento do risco de sangramento clinicamente relevante 9 . 

##### Heparina de Baixo Peso Molecular (HBPM)

 Existem dúvidas quanto às evidências de que as HBPMs possam ser efetivas na prevenção do TEV relacionado aos voos de longa duração 9 . O estudo LONFLIT-3 sugere que o risco de TEV pode ser praticamente abolido com o uso de HBPM 1 , mas não existe comprovação para o seu uso indiscriminado e universal nessa situação 15 . De forma geral, a farmacoprofilaxia com HBPM em VA de longa duração deve ser direcionada aos passageiros considerados de alto risco para o desenvolvimento de TEV, e a avaliação quanto à necessidade de fazê-la ou não deve ser decidida após a mensuração do risco e do benefício de cada passageiro individualmente 11 ^,^ 15 . A enoxaparina na dose de 1 mg/kg, por via subcutânea, duas a quatro horas antes do embarque, pode reduzir o risco de TEV em VA de longa duração 3 . Existem evidências indiretas do aumento do risco de sangramento com o uso de farmacoprofilaxia do TEV em VAs de longa duração 6 . Além disso, deve-se levar em consideração o custo financeiro do uso de anticoagulantes na profilaxia do TEV nesses passageiros 6 . 

##### Anticoagulantes Orais Diretos (DOACSs)

 O uso de DOACSs na farmacoprofilaxia do TEV relacionado às VA de longa duração merece algumas considerações. Esse grupo de medicamentos, que inclui os inibidores diretos do fator Xa (no Brasil, rivaroxabana, apixabana e edoxabana) ou da trombina (no Brasil, dabigatrana), tem algumas características relevantes e que possivelmente irão modificar as condutas profiláticas atuais, conforme os estudos de dados de vida real sejam publicados. As suas meias-vidas curtas, seus rápidos inícios de ação e as suas posologias por via oral podem torná-los as medicações de escolha em um futuro relativamente próximo. Porém, ainda não foram realizados estudos que confirmem a eficácia e a segurança desses medicamentos na profilaxia do TEV em voos de longa duração 1 . 

## CONCLUSÃO

 Ainda existem muitas dúvidas com relação a qual perfil de passageiros poderia se beneficiar, de fato, de medidas profiláticas contra o desenvolvimento de TEV relacionado às VAs de longa duração. Além disso, existe uma falta de consenso universal com relação a quais medidas comportamentais, físicas ou farmacológicas poderiam ser adotadas para proteger cada passageiro. Aliado a isso tudo está o fato de sequer haver uma definição epidemiológica quanto a qual episódio de TEV pode estar relacionado aos voos de longa duração e a partir de quantas horas podemos definir um voo como de longa duração. Portanto, todo passageiro deve ser avaliado individualmente, pesando os riscos e os benefícios da adoção de cada uma das medidas para a profilaxia do TEV em voos de longa duração.
